# Experience on healthcare utilization in seven administrative regions of Tanzania

**DOI:** 10.1186/1746-4269-8-5

**Published:** 2012-01-27

**Authors:** Edmund J Kayombo, Febronia C Uiso, Rogasian LA Mahunnah

**Affiliations:** 1 Institute of Traditional Medicine, Muhimbili University of Health and Allied Sciences, P. O. Box 65001, Dar-es-Salaam, Tanzania

**Keywords:** Tanzania, health care, practitioners, traditional and conventional medicine

## Abstract

Health care utilization in many developing countries, Tanzania included, is mainly through the use of traditional medicine (TRM) and its practitioners despite the presence of the conventional medicine. This article presents findings on the study that aimed to get an experience of health care utilization from both urban and rural areas of seven administrative regions in Tanzania. A total of 33 health facility managers were interviewed on health care provision and availability of supplies including drugs, in their respective areas. The findings revealed that the health facilities were overburden with higher population to serve than it was planned. Consequently essential drugs and other health supplies were available only in the first two weeks of the month. Conventional health practitioners considered traditional health practitioners to be more competent in mental health management, and overall, they were considered to handle more HIV/AIDS cases knowingly or unknowingly due to shear need of healthcare by this group. In general conventional health practitioners were positive towards traditional medicine utilization; and some of them admitted using traditional medicines. Traditional medicines like other medical health systems worldwide have side effects and some contentious ethical issues that need serious consideration and policy direction. Since many people will continue using traditional/alternative medicine, there is an urgent need to collaborate with traditional/alternative health practitioners through the institutionalization of basic training including hygiene in order to improved healthcare in the community and attain the Millennium Development Goals by 2015.

## Introduction

Health care utilization in many developing countries including Tanzania is mainly through the use of traditional medicine (TRM); due to limited financial resources to cover conventional medicinal drugs, facilities and medical personnel. Also conventional health facilities are not available in most rural areas and urban slums [[1,2] and [3]]. Traditional medicine refers to health practices, approaches, knowledge and beliefs incorporating plant, animal and mineral based medicines, spiritual therapies, manual techniques and exercises, applied singularly or in combination to treat, diagnose and prevent illnesses or maintain well-being. The knowledge of traditional medicine has developed over generations within various societies before the era of conventional medicine [4]. However in recent years complementary/alternative medicine (CAM) practices have also emerged such as Homeopathy, Radionic, Magnetic and Chiropractic therapy. Complementary and Alternative Medicine practices may incorporate or base themselves on traditional medicine, folk knowledge, spiritual beliefs, or other approaches to healing [5]

Despite the presence of conventional medicine, referred as scientific medicine, literature reviewed show that TRM is widely used and a rapidly growing health care system world wide. In Africa, for example, up to 80% of the population uses TRM to help meet their health care needs [2,3]. In Asia and Latin America, populations continue to use TRM as a result of historical circumstances and cultural beliefs. In China, TRM accounts for around 40% of all health care delivered [2,3]. In Tanzania current statistics show 60-70% of the population seek healthcare to practitioners from TRM [6,7] and more than 50% of child deliveries are attended by traditional birth attendants (TBAs) and relatives [3,8,9].

There is also an increasing number of people using TRM from 427 million to 629 million far exceeding the total number of visits to primary health care centers [[10-13] and [14]]. These statistics supports the World Health Organization (WHO) argument that considers TRM as one of the surest means to achieve total health care coverage of the world's population [1,2]. The challenge however, lies in the risks and ethics of using traditional medicines that are not systematically documented, authenticated and neither their activity verified or established.

Even though TRM is being widely used in rural areas and urban slums where conventional health facilities, drugs and staff are limited [15,16], some modern scientists and conventional health practitioners consider methods of traditional knowledge and the health care as primitive and with no science involved at all [15,16]. Consequently some conventional health practitioners and scientists continue to shun away from traditional health practitioners (THPs) [[15,16] and [17]], despite contribution made by TRM towards meeting the basic health needs of the rural population in developing countries [18,19]. Limited funds are allocated for carrying studies on TRM practice and scaling up its development compared to conventional medicine [19,20]. However, recent progress in the fields of environmental sciences, immunology, medical botany and pharmacognosy have led researchers to appreciate in a new way the precise descriptive capacity and rationality of various traditional taxonomies as well as the effectiveness of the treatments employed [21,22].

Despite the marginalization of TRM, use of the practice has expanded globally and the practice has gained popularity especially on degenerative diseases and HIV/AIDS in the last decade [21,22]. Health is both biological and cultural, and thus to have a health body, treatment should be holistic; TRM and CAMs qualify for these attributes. Currently there is advocacy by WHO and governments to promote the application of TRM and CAMs health care systems especially on degenerative diseases and HIV/AIDS. This initiative has stirred new investments and design of programs in TRM and CAMs in a number of developing countries [[2,3,21] and [22]]. Hopefully this new impetus will also dispel and address the main concerns on ethics of THPs and risks of TRM and CAM to patients, given that not all the practices or the medicines are known or systematically documented.

Worldwide conventional medicine is officially recognized as the main medical system for provision of healthcare [23]. It is in this system where governments and donor funding is channeled towards improvement of the healthcare system [19]. Notwithstanding, most developing countries have limited financial resources for buying drugs and other medical supplies. Further qualified medical personnel and most of the health supplies are not available [[24,25] and [26]]. Ideally, there should, at least, be a primary health centre within a five kilometer radius [[2,19,24-26] and [27]].

Developing countries have begun to realize that their current health systems are dependent upon technologies and imported medicine that end up being expensive and whose supply is erratic [21,23]. Tanzania for example has attempted to develop a comprehensive conventional healthcare system going right down to the village level [19]. At the village level there are village health posts followed by dispensaries and health centers. These are coordinated at a district and regional level by district and regional hospitals. Normally health centers are bigger units than dispensaries, although some dispensaries might be carrying out activities equivalent to a health centre. Health centers serve a minimum of 3 villages with a population of 50,000 people, whereas a dispensary serves 25-26,000 people [18,19]. Health centers coordinate all dispensaries and health posts within their catchment area. Health centers deliver all basic health services including maternal and child health, minor operations and most carry out HIV testing and counseling. However, these comprehensive plans are compromised by the poor transport system. There are evidences that in most developing countries, roads are impassable and the transportation system is chaotic [24]. Thus, when a person decides to seek medical attention, it may take days to reach the health care facility. Indeed there are pathetic situations where patients are brought to the hospital on wheel barrows, bicycles, on donkeys or physically carried on stretchers [24]. All these incidences contribute to the reported high maternal and infant mortality rate in many developing countries.

In response to this pathetic situation in the last 30 years, there has been continuous urge of building or increasing health care institutions and primary health care activities in developing countries as a way of improving health care deliveries especially in rural areas and urban slums. The questions that remain unanswered and the subject of this study are: Have these efforts increased healthcare provision including medical personnel and other medical supplies? What services are provided at health facilities? Have these efforts resulted in any change on utilization of traditional medicine? What is the general attitude of health workers towards traditional medicine? Is there any perceived risk in taking traditional medicines; and what are the ethical issues surrounding TRM provision? The other pertinent question is means of transport for both healthcare providers and patients.

This study aimed to answer the raised questions by carrying a survey of health workers in both rural and urban areas of Tanzania focusing on:

i. Healthcare coverage by a health centre

ii. Use of traditional medicine in the treatment/management of diseases and ill health conditions in the community

iii. Attitudes of conventional health workers towards the use of traditional medicine

iv. Perceived risks and ethics on the use of traditional medicine

v. Attitude towards collaboration with THPs in provision of health care

vi. Means of transport for both health workers and patients

## Research methodology

The study used qualitative approach in data collection and this was done in seven (33% of the total administrative regions of Tanzania) regions of Tanzania namely Arusha, Coast, Dares-salaam, Iringa, Kilimanjaro, Morogoro and Tanga in the period 2004-2007. The selected regions have 42 districts in total and of these 14 (13% of total number of districts in the country) districts were selected for this study. The total number of people in the selected regions was 14,173, 740 and of these 51% were female (Estimated from 2002 Population Census) [28]. These regions and districts were purposively selected due to their proximity to Dar-es-Salaam, accessibility to the district headquarters and the time allocated to the project. The main socio-economic activities of the people living in the selected districts is peasantry farming for both food and cash crops and as well as livestock keeping mainly in the Arusha region.

The study targeted health centres, a level that supervises dispensaries and health posts; hence information at the lower level can also be provided by these centres. However, in some areas dispensaries were purposefully selected when they served a more or less equal number of people as the health-centres. The study population on the other hand was mainly the Health-centre Managers. The managers of health centres and dispensaries as administrators of the health facilities are well versed with issues related to the provision of healthcare in the country. These managers are mostly trained at the level of Medical Assistants and few are degree holders. In total 33 health facility managers were interviewed.

About 90% of the health centres and dispensaries were based in rural areas. The data collection was face to face in-depth interview conducted by the researchers. The interview sought information on the following key issues:

• Distribution of health facilities within their respective service areas

• Utilization of traditional medicine and their attitudes

• Perceived risk and ethics on the use of traditional medicine

• Willingness to collaborate with traditional medicine system

• Means of Transport

Qualitative sociological and anthropological methods were used for data analysis where codes were designed and allocated as shown in Grounded Theory Procedures and Techniques [29]. In the process of analyzing the information, axial coding were used where the data were put according to the selected categories and subcategories; in this way making connection between the central idea and between category and subcategory. The managers of health centres and dispensaries rated the standard of hygiene in their facilities. The scale given was between 1- 5 where 1 = very low and 5 = very high.

## Results

### Coverage of the Study Area

A total of 33 respondents from 20 health facilities aged between 30-50 years old were interviewed and of these 60% were females. The findings showed that, each health centre covered an area that ranged from 130-1200 Km^2 ^with total number of villages served ranging from 2-25 villages per health facility.

Health centres covered per region and with the respective number of villages served by these centres are shown in Table 1. Fifteen percent of health centres served more than 10 villages. The findings showed that number of households per health centre ranged from 800-14,770; and each household had 8-10 people. Health facilities served in total 117,160 to 147,700 people as calculated from the number of household per health centre. Private health facilities operating within health centre coverage ranged from 0 to 10 and 80% of these facilities were in urban districts and municipal centres.

**Table 1:** Number of Villages Served by Health Centre

	Number of Villages Served by Health Centre		
**Region/District**	**1-5**	**6-10**	**Above 10**

**Dar-es-Salaam**			
- Ilala	1	1	**-**
- Kinondoni	-	2	-
- Temeke	1	-	-

**Arusha**			
- Arusha Urban	1	-	-
- Arumeru	-	2	-

**Coast**			
- Kisarawe	1	1	-

**Iringa**			
- Iringa	1	-	-

**Kilimanjaro**			
- Mwanga	1	-	-
- Moshi Urban	-	1	-
- Moshi Rural	1	-	-

**Morogoro**			
- Morogoro Rural	-	-	1
- Mvomero	2	-	-

**Tanga**			
- Tanga rural	1	-	1
- Tanga Municipal	-	-	1

**Total**	10	7	3

### Services Provided by Health Centres

The findings showed that all the surveyed health centres and dispensaries provided basic medication for various diseases; carried out minor operations and laboratory services. Voluntary counseling and testing for HIV/AIDS including family planning services were provided in 75% of the health centres. All health facilities had all basic materials for maternal health, antibiotics, bandage materials, panadol, fancidar, mebendezole and disinfectants. However, all the facility managers informed that drugs and medical materials were supplied monthly and that these supplies were enough for only the first two weeks of the month. One of the managers of the health facilities remarked and said,

"*We are facing a serious shortage of drugs and medical supplies from the third and fourth week of the months because most of the drugs and medical supplies are only enough for the first two weeks of the month"*

They rated the standard of hygiene of their facilities fairly good; between 3 and 5 ranges. However, an independent view could not be obtained to corroborate their rating.

### Means of Transport

In most developing countries roads are impassable and transportation system is chaotic [24]. In the surveyed rural areas, roads were poorly developed and no reliable bus transport system was operational within the district. The MCH of one of the health centers remarked,

"*It becomes worse during the rainy season when going out for outreach service; and some of the areas are not accessible"*

Thus health workers and patients walked to and from the respective health facility. The means of transport for outreach services like supervision, outreach care and drug distribution to the dispensaries, health posts, during health campaign and vaccination of children were bicycle (30.3%), walking by foot (27.3%) and others as shown in Table 2.

**Table 2:** Means of Transport for Health Workers for Outreach Services

Means of Transport	Number of respondents	Percent (%)
Official Vehicle	5	15.1

Bicycle	10	30.3

Motor Bike	3	9.1

Public Transport	6	18.2

On foot	9	27.3

Total	33	100

### Utilization of Traditional Medicine

Thirty percent of the respondents were either new or recently employed and had no opportunity to interact with people in the communities to give an opinion on the ratio of usage of traditional medicine. The rest indicated that TRM was being used even in places that were near the health facilities especially for child deliveries. One of the district medical officers in our study remarked,

"*Many pregnant women residing around the district hospital are being assisted by traditional birth attendants at delivery"*

The findings have also revealed 50% of the conventional health workers have in one way or another used traditional medicine for various ailments. The findings also revealed that the number of traditional medicine practitioners working within health centres service radius ranged from none to 10 and the number of TBAs ranged from none to 152. However, no traditional medicine clinics were identified, given that most THPs and TBAs conduct their treatment in their respective living premises with no defined working place that can serve as a clinic. Most health centre managers indicated that traditional health practitioners (THPs) treated a wide range of diseases as shown in Table 3.

**Table 3:** Diseases/Illnesses Reported to be Managed by THPs

Disease/Illnesses	Number of responses (n = 62)	Percent (%)
Mental illness (epilepsy, psychosis, Schizophrenia)	11	17.7

Family planning and menstrual disorders	5	8.1

Stomachache disorders, dysentery, diarrhea and abdominal pain	5	8.1

Stroke and hypertension	5	6.4

Diabetes	4	9.6

HIV/AIDS and sexual transmitted diseases	6	8.1

Malaria and febrile convulsion	5	8.1

Asthma and cough	5	8.1

**others	16	25.8

Total	62	100

* Multiple responses were allowed

** others included snake bite, cancer, toothache, skin diseases, anemia, born fractures, fresh wounds and ulcers, rheumatism, migraine, headache and flue

### Diseases/illnesses conditions treated by THPs

Table 3 shows that THPs are managing a number of health conditions. The findings suggest that mental illness (17.7%) and HIV/AIDS (9.6%) were the leading conditions being managed by THPs. One of the managers reported,

"*THPs are managing some of the HIV/AIDS symptoms such as diarrhea, herpes zoster, fungus and are also good on mental health"*

Other health problems managed by THPs include family planning, stomach ache, stroke and hypertension, abdominal disorders, malaria, febrile conditions and asthma. Each of these conditions scored more than 8% of the responses.

### Attitude of Health Workers towards Traditional Medicine

The findings on attitude of health workers towards traditional medicine showed that 50% of the respondents had knowledge of TRM. They considered it effective on certain diseases. Some health workers reported to be advising patients to use common remedies mainly as first aid items such as honey, lemon grass, drumstick (*Moringa oleifera*), aloe species and others. Some respondents who were new to the study area or recently employed could not form an opinion on what percentage of their respective population used TRM. In general there was no mention of referring patients to THPs from health workers; but health workers were receiving patients from THPs. However, there is no established referral system either. It was observed that 50% of the surveyed health centres did not collaborate with any THPs. None of the conventional health workers had ever visited THPs' premises to assess their practice or hygiene including sharing knowledge on health management of various health problems affecting their respective community.

### Health Related Risks associated with Use of Traditional Medicine

Traditional medicine has been associated with many risks including maternal and early infant deaths [26]. The findings on health-related risks associated with TRM was synthesized and grouped into two main categories. The first are risks associated with patient handling and diagnosis and the second are the ones associated with the practice and treatment. In the first category the following were reported:

i. Handling and mismanagement of accident victims that lead to delay in getting proper treatment

ii. Child fevers and malaria are mostly confused with traditional beliefs and superstitions associated with witchcraft.

iii. Improper diagnosis/management based only on presenting symptoms without laboratory support leads to delayed treatment

iv. Patients are sometimes misadvised by THPs due to their low scientific/technical health related knowledge

In the second category the following were reported:

i. Labor enhancing medicinal plants that leads to delivery complications and sometimes rapture of the uterus

ii. Mixed treatment of both TRM and allopathic medicine that might lead to adverse and toxic effects for contraindicated medicines.

iii. Use of amulets for young children that can lead to diarrhea due infant sucking the amulet.

iv. Vapor inhalation through steam bath can lead to bronchial asthma

v. Dehydration caused by use of herbal purgatives and emetics

Notwithstanding none of these perceived risks had been documented with recorded patient incidences in any of the health facilities where the study was done.

### Ethical issues

Ethical issues are among of the major concerns in provision of traditional medicine healthcare. This study identified five main ethical issues on TRM as viewed by the health workers and these were:

i. Delayed referral of patients to conventional health care

ii. Harmful practices such as application of animal and human excreta, massaging practices that inflict burns from either hot stone or steam bath treatment and urinating on convulsing patient.

iii. Diagnostic practices that do not respect privacy of the patients especially on divination and mental health rituals

iv. The money economy has lead some healers to demand high prices that result in treatment denial

v. Provision of sub doses by THPs in order to make more money or retain clients

Conventional health workers expressed the following opinions on how the identified ethical issues could be handled:

i. Training and collaboration between conventional and traditional health practitioners to enable THPs to acquire knowledge and skills to identify dangerous signs and symptoms to make proper prompt decision on patient's health.

ii. Sensitize and create awareness on ethical issues to THPs, conventional health workers and the community

iii. There should be a policy or legislation on TRM practice to regulate ethical issues and eliminate quacks and charlatans.

iv. Establish proper pricing system and prescription of traditional medicine.

Other comments regarding the TRM and its practitioners expressed by the conventional health workers are as follows:

a. There is a need to sensitize conventional health workers to interact with practitioners of TRM

b. Need to organize frequent seminars to address TRM practice and medicinal plants at health centre level

c. Need to develop a TRM training manual and procedures for handling HIV/AIDS patients.

d. Establish a formal feedback system on health care.

### Collaboration and Integration

Collaboration between the two medical systems has to first address a major challenge since the traditional and conventional medicine systems differ in their perception and understanding of diseases causation and treatment. The research aimed on getting views on the following four pre-identified areas: i) Integration of traditional medicine practice into conventional health care system, ii) Traditional health practitioners to work with conventional health workers to develop treatment dosages, iii) Conventional health workers and THPs to establish joint treatment clinics and iv) Training conventional health workers to acquire knowledge on medicinal plants (MPs) and TRM practice. The conventional health workers were asked to indicate at least two areas of collaboration and integration from the four suggested areas. Table 4 shows that health workers would first prefer to be trained on knowledge of MPs and TRM (33.3%) and the issue of establishing joint treatment clinics was rated lowest (17.9%).

**Table 4:** Proposed Areas for Collaboration and Integration

What is to be done first	Number of responses	Percent (%)
Integration of traditional practice into the general health care system	10	27.7

Traditional healers should work with modern doctors to come up with dosage	9	23.1

Health workers and traditional healers to support each other and by having a joint clinic	7	17.9

Training health workers to acquire knowledge of MP and TRM	13	33.3

Total	39	100

Two responses were allowed

## Discussion

This study has shown that most health facilities served more than 10 villages implying that they were serving more than the expected or planned capacity. Health centres are expected to manage 50,000 people, but the present findings showed that on average they served between 117,160 to 147,700 people. Private health facilities are expected to reduce the workload of the public sector; however, most of them are centered in urban centres where potential clients/patients are available. Shortage of health facilities is a common phenomenon in all developing countries [[15,19,24] and [26]] and the most hit areas are the rural people of African countries south of the Sahara [[15,24,26] and [30]].

This study has shown that drugs and other medical supplies were provided monthly and these lasted for the first two weeks of the month. This suggests that drugs and other medical supplies were limited for the next two weeks of the month. These findings underscores Muhondwa et al [31] study in Mtwara District and Kitula [32] in Udzungwa Mountains Forests Morogoro region who have reported that drugs and other supplies were available only in the first two weeks of the month. Similar results have been reported by studies reviewed in Africa [[3,15] and [32]] and in developing countries in general [24,25]. For example Disease Control Priorities Project [33]; Rahman et al [25] and Chudi [24] show that nearly one-third of the world's population lacks access to modern drugs and vaccines, and the most hit are the countries south of the Sahara. The majorities of these people are extremely poor and live in remote rural areas.

Transport is essential for patients' movement and distribution of drugs, blood and other supplies to health facilities [24]. In this study transport was identified as the major concern and constraint during supervision and vaccination. The main means of transport was either by bicycle or on foot (see Table 2). It is most likely that some of the areas are not reached during supervision, outreach services and during child vaccination especially during the rainy seasons. The present findings explain why many child bearing women deliver at home with assistance of close relatives and traditional bath attendants [[10,30,34] and [35]]. In general all these reduce poor people's ability to access preventive, curative, and emergency health services and can result in lower health status and high mortality rates as being experienced in Tanzania and other developing countries. Given this poor transport system; training of THPs is very important, so that they could provide better health care services at the community in a standardized manner. This will help most regions of the developing world reach the Millennium Development Goals (MDGs) for Health for all by 2015.

The present findings have underscored the claim that many people use TRM for various ailments (see Table 3, 4). However, this information does not reflect a true picture due to the limited number respondents and some being new in the working stations. There is need to carry out a larger survey of health facilities. In general health workers confirm that there are some ill health conditions that TRM practitioners manage better than conventional medicine especially on mental health problems and some clinical symptoms associated with HIV/AIDS. The use of traditional medicine and the popularity of complementary and alternative medicine amongst the general population seem to be increasing world wide [[10,11] and [22]]. Consumer pressure is considered to be the major driving force behind this growth [23].

It is well documented that there is no health system in the world that has no side effects from its practice or its remedies [23]. Generally conventional health workers consider the use of TRM to have several side effects; however, no specific cases were recorded within the health centres surveyed in this study. Besides the potential side effects, the present study has identified unethical practices that need to be addressed. In Chinese medicine animal and human excreta is used after being processed [36,37]. However, similar processing is not reported in the case of Tanzania. There still exists mistrust between the traditional and conventional health care system. Referral between the two systems of health would have been an innovative strategy to promote mutually-beneficial collaboration [13]. Health workers had the opinion that both THPs and the conventional health workers should be given special training on how the two health systems work in provision of healthcare. Two probable suggestions can be derived. First the need to know materials used in traditional medicine and determine active ingredients is driven by the bio-prospecting approach and need to identify more effective remedies for use in conventional medicine. However, not every remedy used in TRM can be verified in the laboratory. For example the herbal remedies used for ritual treatment may be difficult to find active ingredients. Second, the intrinsic drive to seek more knowledge for individual use and/or practice is at play.

Effective collaboration needs trustworthiness, transparency, building mutual respect, sharing knowledge [7,22] and this will gradually lead to knowing medicinal plants and traditional medicine. These kinds of interactions and regular meeting for exchanging ideas on management of health problems are essential for effective collaboration. Tanzania has enacted a Traditional and Alternative Medicine Act 2002 that legalises the practice of TRM; and the two health care systems are now under one ministry. Thus the two are now better placed to design a framework on areas of collaboration to meet the goal of better health for all the people by year 2015.

## Conclusion

The present study shows that, most people in rural areas and urban slums have limited access to conventional medicine. This is compounded by poor transport system to health facilities and outreach services. It is generally assumed that TRM is more accessible and available in most areas. The major concern on the use of traditional medicine is the probable side effects and some perceived unethical practices by the THPs. There still exists a big mistrust between the practitioners of the two health care systems. Limited resources for training adequate conventional medical practitioners and equip the health facilities with necessary supplies for provision of quality health care to the community will compel many Tanzanians and other resource poor countries to continue using traditional medicine. Collaboration between the two health systems will improve health care provision and increase safety. Traditional and conventional health practitioners should be seen as partners in provision of healthcare.

## Competing interests

The authors declare that they have no competing interests.

## Authors' contributions

This study was part of the East African Network on Traditional Medicinal Plants and Traditional Medicine. The study was conceived by the East African Research team that included researchers from Uganda, Kenya and Tanzania. In Tanzania the researchers who participated in design and coordination were RLAM and FCU. The study area was divided into three zone; and this included Dar-es-Salaam and Coastal region; the second zone included Arusha, Kilimanjaro and Tanga; whereas zone three included Morogoro and and Iringa. EJK collected data from zone one, FCU collected data from zone one and three; whereas RLAM collected data from zone two and three. The three researchers compiled the raw data, transcribed, analyzed and summarized this data in the format as it appears in this manuscript. The researchers worked as one team all the process of revision and each has read the final draft and approved the manuscript.

## Authors' information

The authors of this manuscript are all Tanzanian citizen working in the Institute of Traditional Medicine of Muhimbili University of Health and Allied science, Dar-es-Salaam, Tanzania
